# Evolutionary Conservation of the Components in the TOR Signaling Pathways

**DOI:** 10.3390/biom7040077

**Published:** 2017-11-01

**Authors:** Hisashi Tatebe, Kazuhiro Shiozaki

**Affiliations:** 1 Nara Institute of Science and Technology, Graduate School of Biological Sciences, Ikoma, Nara 630-0192, Japan; kaz@bs.naist.jp; 2 Department of Microbiology and Molecular Genetics, University of California, Davis, CA 95616, USA

**Keywords:** target of rapamycin (TOR), kinase, GTPase, signaling, TORC1, TORC2, RHEB, RAG

## Abstract

Target of rapamycin (TOR) is an evolutionarily conserved protein kinase that controls multiple cellular processes upon various intracellular and extracellular stimuli. Since its first discovery, extensive studies have been conducted both in yeast and animal species including humans. Those studies have revealed that TOR forms two structurally and physiologically distinct protein complexes; TOR complex 1 (TORC1) is ubiquitous among eukaryotes including animals, yeast, protozoa, and plants, while TOR complex 2 (TORC2) is conserved in diverse eukaryotic species other than plants. The studies have also identified two crucial regulators of mammalian TORC1 (mTORC1), Ras homolog enriched in brain (RHEB) and RAG GTPases. Of these, RAG regulates TORC1 in yeast as well and is conserved among eukaryotes with the green algae and land plants as apparent exceptions. RHEB is present in various eukaryotes but sporadically missing in multiple taxa. RHEB, in the budding yeast *Saccharomyces cerevisiae*, appears to be extremely divergent with concomitant loss of its function as a TORC1 regulator. In this review, we summarize the evolutionarily conserved functions of the key regulatory subunits of TORC1 and TORC2, namely RAPTOR, RICTOR, and SIN1. We also delve into the evolutionary conservation of RHEB and RAG and discuss the conserved roles of these GTPases in regulating TORC1.

## 1. Introduction

**Figure 1:**
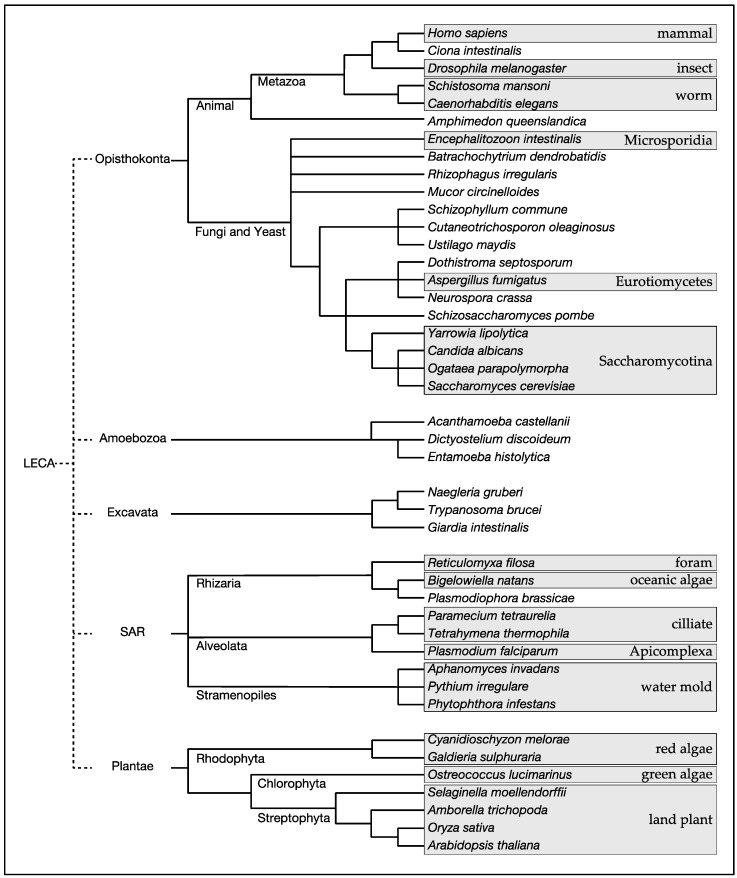
A consensus cladogram of the five major eukaryotic clades with selected eukaryotes. The cladogram was constructed based on the proposed phylogenetic relationships in [4]. LECA: last eukaryotic common ancestor.

Target of rapamycin (TOR) is a phosphoinositide-3 kinase-related protein kinase that plays pivotal roles in controlling a wide variety of cellular processes in response to a broad spectrum of intracellular and extracellular stimuli [1]. TOR was first identified through a genetic screen for budding yeast mutants that are resistant to the immunosuppresant rapamycin [2]. Subsequent identification of TOR in humans and other species revealed evolutionary conservation of TOR from yeast to humans [3]. Expanding genome data of diverse species have revealed that TOR exists ubiquitously in eukaryotes of all five major clades, Opisthokonta (animals, yeast and fungi), Amoebozoa (protozoa), Excavata (protozoa), SAR (protozoa, brown algae), and Plantae (red algae, green algae, land plants) [4] (Figure 1; Table 1). Among the eukaryotic species, notable exceptions are obligate intracellular parasites, such as *Plasmodium falciparum* that belongs to the phylum Apicomplexa in the SAR clade and *Encephalitozoon intestinalis* that belongs to the phylum Microsporidia in the fungal kingdom [5] (Table 1). Thus, it is likely that TOR arose in the last eukaryotic common ancestor (LECA) and has stayed vital in all eukaryotes except those exclusively living in an extremely stable environment such as the inside of host cells.

**Figure 2:**
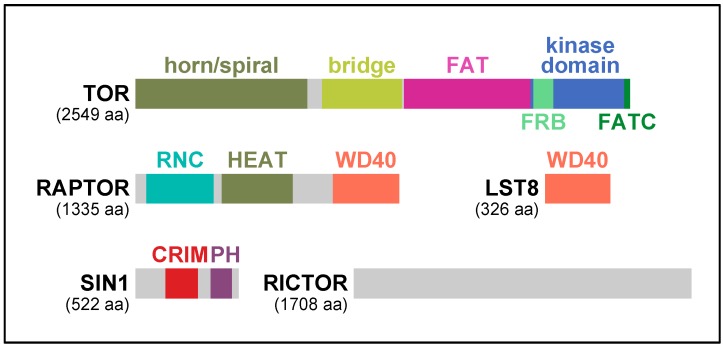
Domain structure of the subunits of the TOR complexes in human. TOR: target of rapamycin; RAPTOR: regulatory associated protein of mTOR; LST8: lethal with sec thirteen 8; SIN1: stress-activated protein kinase interacting protein 1; RICTOR: rapamycin-insensitive companion of mTOR; FAT: FRAP–ATM–TRAPP; FRB: FKBP-Rapamycin Binding; FATC: FAT C-terminus; RNC: RAPTOR N-terminal conserved; HEAT: Huntington-EF3-PP2A-TOR1; CRIM: conserved region in the middle; PH: pleckstrin homology.

TOR kinase possesses multiple characteristic motifs and domains: α-helical HEAT (Huntington-EF3-PP2A-TOR1) repeats, the FRB (FKBP-Rapamycin Binding) domain, the FAT (FRAP–ATM–TRAPP) domain, the FATC (FAT C-terminus) domain, and the phosphoinositide-3 kinase domain (Figure 2). In the crystal structures of the C-terminal half of human TOR, the kinase domain is in an enzymatically active conformation [7]. However, the active site is located at the bottom of the deep catalytic cleft and sterically hindered by the surrounding structural elements, where multiple hyper-activating mutations of TOR have been identified [7]. Thus, it has been proposed that activity of TOR is controlled primarily by restricting active-site access [7]. Recent cryo-electron microscopy (cryo-EM) analyses of human and fungal TOR also revealed that the N-terminal HEAT repeats of TOR form two solenoid structures: a highly curving structure called “horn” or “spiral” and a less curving structure called “bridge” [8,9,10] (Figure 2). Two molecules of TOR shape a two-fold symmetry ring by physical contact between the “horn/spiral” and the “bridge” [8,9,10]. Considering the significant homologies throughout the N-terminal HEAT repeat region of TOR, it is expected that the characteristic solenoid structures as well as the two-fold symmetric ring formation are very common among TOR orthologs.

**Figure 3:**
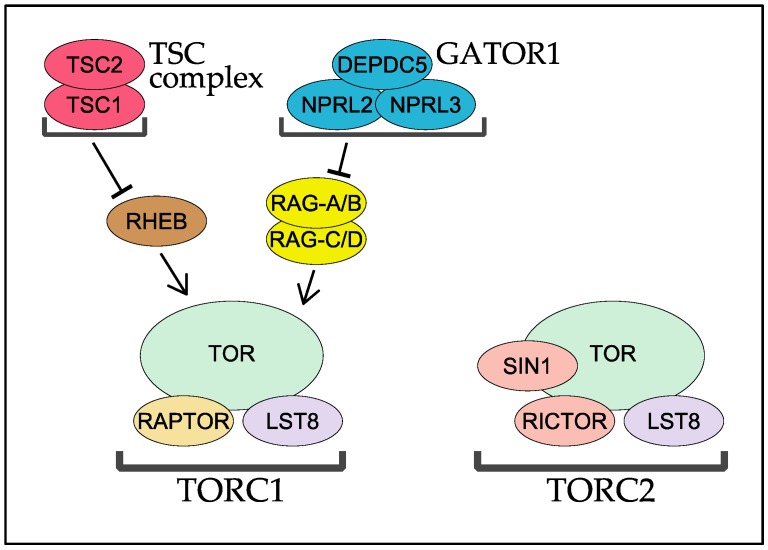
Composition and regulation of TORC1 and TORC2 in human. DEPDC5: DEP domain containing 5; GATOR1: GAP activity towards RAGs 1; NPRL2: nitrogen permease regulator 2-like protein; NPRL3: nitrogen permease regulator 3-like protein; RHEB: Ras homolog enriched in brain; TORC1, 2: TOR complex 1, 2; TSC1, 2: tuberous sclerosis complex 1, 2.

TOR forms two functionally and structurally distinct protein complexes TOR complex 1 (TORC1) and 2 (TORC2), of which only TORC1 is sensitive to rapamycin [11] (Figure 3). TORC1 inhibition by rapamycin is mediated by physical binding of rapamycin and the peptidyl-prolyl cis-trans isomerase FKBP12 to the FRB domain of TOR kinase [11]. Among eukaryotic species, TORC1 contains the TORC1-specific regulatory subunit regulatory associated protein of mTOR (RAPTOR) and TORC2 with two TORC2-specific subunits, rapamycin-insensitive companion of mTOR (RICTOR) and stress-activated protein kinase interacting protein 1 (SIN1) (Figure 3). The two TOR complexes share the same catalytic subunit TOR kinase and a regulator subunit called LST8 (Figure 3), and the physiological and biochemical distinction of the two complexes is mainly determined by the complex-specific regulatory subunits. Below, we summarize and discuss the evolutionarily conserved molecular functions of RAPTOR, RICTOR, and SIN1 subunits, with emphasis on their structures. Since the small GTPases Ras homolog enriched in brain (RHEB) and RAG have been emerging as critical regulators of mammalian TORC1, we also review the molecular functions and evolutionary conservation of these small GTPases and their regulators [1,12]. The evolution of nutrient-sensing pathways regulating TORC1 is also discussed in a recent review article [13]. Note that, throughout this review, we utilize human protein names without the prefixes “m” (for mammal) or “h” (for human) to describe each component in the TOR signaling pathways [14].

## 2. RAPTOR, the Signature Subunit of TORC1

When TORC1 was first discovered in yeast and mammals, RAPTOR was identified as the signature subunit of TORC1 [11,15,16,17]. Except for ciliates, such as *Paramecium tetraurelia* and *Tetrahymena thermophile*, RAPTOR is found in every eukaryotic species that possess functional TOR kinase (Table 1) [5]. Therefore, it is presumed that RAPTOR arose with TOR in LECA and has been conserved during the evolution of eukaryotes. In its primary structure, RAPTOR has several characteristic segments. At the N-terminus is a region highly homologous among RAPTOR orthologs, and thus called the RAPTOR N-terminal Conserved (RNC) domain [8,15] (Figure 2). In the ternary structure of human and fungal RAPTOR, RNC forms a caspase-like fold with several extra helices [8,10]. The extra helices are tightly packed and placed between the caspase-like fold and the α-solenoidal HEAT repeat structure lying in the middle of RAPTOR [8,10] (Figure 2). At the C-terminus is a WD40 repeat domain that shapes a seven-bladed β-propeller [8,10,15,16] (Figure 2).

RAPTOR plays multiple essential roles in TORC1, including assembly and stabilization of the complex as well as substrate recognition. According to the recent cryo-EM studies, the extra helices of RNC and the first several helices of the HEAT repeats together form a wedge to stabilize the interaction between the two α-solenoidal structures “horn/spiral” and “bridge” of TOR [8,10]. Since RNC and the HEAT repeats are highly conserved in the primary sequences of the RAPTOR orthologs, those helical regions should retain similar structural characteristics for the assembly and stabilization of TORC1 among species. Mutations in the WD40 domain also compromise the assembly and stabilization of TORC1 [15,18], but ternary structure of TORC1 does not clearly illustrate how the WD40 domain of RAPTOR is involved in the TORC1 architecture [8,10].

In mammals, p70 ribosomal S6 kinase 1 (S6K1) and eukaryotic initiation factor 4E-binding protein 1 (4E-BP1), two of the best-characterized TORC1 substrates, possess a five amino-acid stretch called TOR Signaling (TOS) motif [19,20]. Through their TOS motifs, RAPTOR physically interacts with S6K1 and 4E-BP1, resulting in their phosphorylation by TORC1. In the cryo-EM structure of human TORC1, the caspase fold of RAPTOR is in proximity to the active site of TOR kinase, implying that the caspase-fold region directly binds the TOS motif to bring the substrates toward the catalytic center of the complex [8,10]. The model is further reinforced by the fact that caspase proteases recognize four-residue sequences ending with aspartic acid, as the fourth position of the five-amino acid-long TOS motifs is occupied by aspartic acid. However, it remains to be experimentally determined if the caspase fold of mammalian RAPTOR physically binds the TOS motif. In addition, not all the mammalian TORC1 substrates have apparent TOS motif-like sequences [21]. It should also be noted that the TOS motif has not been reported in TORC1 substrates of non-animal species including fungi, although the caspase-fold region of RAPTOR is highly conserved also in those species.

## 3. TORC2 and Two Key Regulatory Subunits: RICTOR and SIN1

Rapamycin-insensitive TORC2 is the second complex formed by TOR kinase. TORC2 was first discovered in the budding yeast *Saccharomyces cerevisiae* with RICTOR and SIN1 as its regulatory subunits [11,17], followed by identification of TORC2 in other organisms including mammals [22,23,24]. Currently, TORC2 has been identified in four of the five eukaryotic clades with Plantae being the only exception (Table 1) [5]. Therefore, it is speculated that TORC2 arose in LECA but was lost at the very beginning of the evolution of algae and plant. It is also conceivable that TORC2 arose after Plantae diverged from LECA, although how the five major clades of eukaryotes diverged during evolution remains unresolved [4].

The TORC2-specific subunit RICTOR plays indispensable roles for the TORC2 function, of which the proper assembly and stabilization of TORC2 appear to be its primary role [25,26]. The amino acid sequence of RICTOR is highly conserved among species [11,24], but unfortunately, no detailed structural information has been available for this essential TORC2 subunit. A chemical crosslinking study of TORC2 in budding yeast demonstrated that the very C-terminus of RICTOR occupies the vicinity of the FRB domain of TOR kinase; indeed, C-terminal truncation of RICTOR is sufficient to make budding yeast TORC2 sensitive to rapamycin [27]. Thus, the C-terminus of RICTOR prevents the rapamycin-FKBP12 complex from binding to the FRB domain of TOR kinase in TORC2, which makes TORC2 insensitive to rapamycin [27].

SIN1 is another conserved regulatory subunit of TORC2 essential for its function. SIN1 was identified in the fission yeast *Schizosaccharomyces pombe* as a protein that interacts with the stress-activated mitogen-activated protein (MAP) kinase Spc1 (also known as Sty1) [28]. Nonetheless, significance of the interaction remains unclear until today [22]. Subsequent identification of SIN1 as a component of TORC2 opened the door for studying the SIN1 function [11,23]. In its primary structure, SIN1 is divided into several discrete regions [29,30,31]. The N-terminal region is homologous only between closely related species but rather divergent among a wide variety of species. The C-terminal region is composed of a PH (pleckstrin homology) domain, a well-known lipid-binding domain that selectively binds phospho-inositides in cellular membranes [32] (Figure 2). The central region is most highly conserved in SIN1 and hence called conserved region in the middle (CRIM) [30] (Figure 2).

In the absence of SIN1, TORC2 is disassembled in budding yeast and animals, suggesting a critical role of SIN1 in the stabilization of TORC2 [23,26,33]. However, the importance of SIN1 in TORC2 assembly may differ among species, as the remaining TORC2 subunits stay associated in fission yeast cells lacking SIN1 [34]. Chemical crosslinking of budding yeast TORC2 revealed configuration of SIN1 in TORC2; the N-terminus of SIN1 is positioned beside the RICTOR subunit, the region N-terminal to CRIM is located next to LST8, and the C-terminal PH domain is near the kinase domain of TOR [27]. Proximity of the SIN1 PH domain to the TOR kinase domain was observed also by electron microscopy [27].

The CRIM domain is a ubiquitin-like domain with a characteristic acidic protrusion [34]. From yeast to humans, the CRIM domain functions as a substrate-recruiting module in TORC2, by directly binding TORC2 substrates in a manner dependent on the acidic protrusion [34,35,36]. The CRIM domain can distinguish the TORC2 substrates, such as human AKT and protein kinase C (PKC), from the TORC1 substrate S6K1, though these kinases all belong to the same AGC family [34]. It remains to be determined how CRIM specifically recognizes the catalytic domain of the certain AGC kinases [34]. The CRIM domain is dispensable for TORC2 assembly because TORC2 is fully assembled with the CRIM-less mutant SIN1 in fission yeast as well as in mammalian cells [34].

The C-terminal PH domain is highly conserved among SIN1 orthologs in diverse species, but its physiological role and significance seem to be somewhat controversial. In budding yeast, the SIN1 PH domain is essential for the TORC2 function [37]. Because of its ability to bind phospho-inositide and localize to the plasma membrane, the SIN1 PH domain has been proposed to target TORC2 to the cell surface of budding yeast [37]. Further corroboration of the model would be possible by introducing point mutations that disable the PH domain for binding phospho-inositide. In fission yeast, the SIN1 PH domain is dispensable for the TORC2 function [34]. Fission yeast TORC2 is also localized at the plasma membrane, but the membrane localization is observed even in mutant cells lacking the SIN1 subunit [38]. In mammals, TORC2 has been observed at various subcellular locations, including the endoplasmic reticulum, mitochondria, mitochondria-associated endoplasmic reticulum membranes, early and late endosomes, and the plasma membrane [39,40,41]. The SIN1 PH domain appears contributing the plasma membrane localization of TORC2 [39,42], although the physiological significance of the PH domain and the plasma membrane localization remains obscure in mammals.

## 4. RHEB and TSC

RHEB is a Ras-like small GTPase essential for TORC1 activity in mammals [43]. Although the precise molecular mechanism is unknown, GTP-bound active RHEB physically binds and stimulates TORC1 activity [44,45] (Figure 3). RHEB is inactivated by its own GTPase activity, which is promoted by GTPase activating protein (GAP) activity of the tuberous sclerosis complex (TSC) protein complex [43] (Figure 3). In mammals, the TSC complex is composed of three subunits TSC1, TBC1D7, and TSC2 [46]. Of these, TSC2 alone is sufficient for the GAP activity toward RHEB *in vitro* [47], while both TSC1 and TSC2 are indispensable for the function of the TSC complex *in vivo* [43]. Multiple physiological stimuli, such as cellular energy status and extracellular growth factors, converge on the TSC complex to regulate the guanine-nucleotide binding state of RHEB [1].

While RHEB is absolutely essential for TORC1 activity in mammals, its requirement appears to substantially vary among Opisthokonta that include mammals as well as insects, worms, filamentous fungi and yeast. RHEB is an essential activator of TORC1 in the fly *Drosophila melanogaster* [48,49]. While TBC1D7 is a vertebrate specific protein, TSC1 and TSC2 co-exist in the fly and function together as GAP for RHEB [50]. RHEB also acts as a positive regulator of TORC1 in *Caenorhabditis elegans* [51]. Both TSC1 and TSC2, however, are absent in the genome of the *Caenorhabditis* species *C. brenneri*, *C. briggasae*, *C. elegans*, *C. japonica*, and *C. remanei* (Table 1; data not shown). Moreover, neither RHEB nor the TSC subunits can be found in the genomes of the worm species *Hymenolepis microstoma*, *Echinococcus granulosus*, *Echinococcus multilocularis*, *Opisthorchis viverrini*, *Schistosoma haematobium*, and *Schistosoma mansoni* (Table 1; data not shown). Therefore, a certain animal species may partially or completely lose RHEB-dependent regulation of TORC1, although the absence of RHEB and the TSC subunits described above is contingent on accurate genome annotations of those species. In the fungal kingdom, RHEB is indispensable for TORC1 activity and cellular viability in the fission yeast *Schizosaccharomyces pombe* [52,53,54], while it appears to play only a limited role in the viability and virulence of *Aspergillus fumigatus* [55]. Interestingly, this pathogenic fungus as well as *Cladophialophora bantiana* and *Trichopyton equinum* is a member of the class Eurotiomycetes (Table 1), in which all the nineteen species we examined lack TSC1 (Table 1; data not shown), implying unique function and regulation of RHEB in this class of fungi.

**Figure 4:**
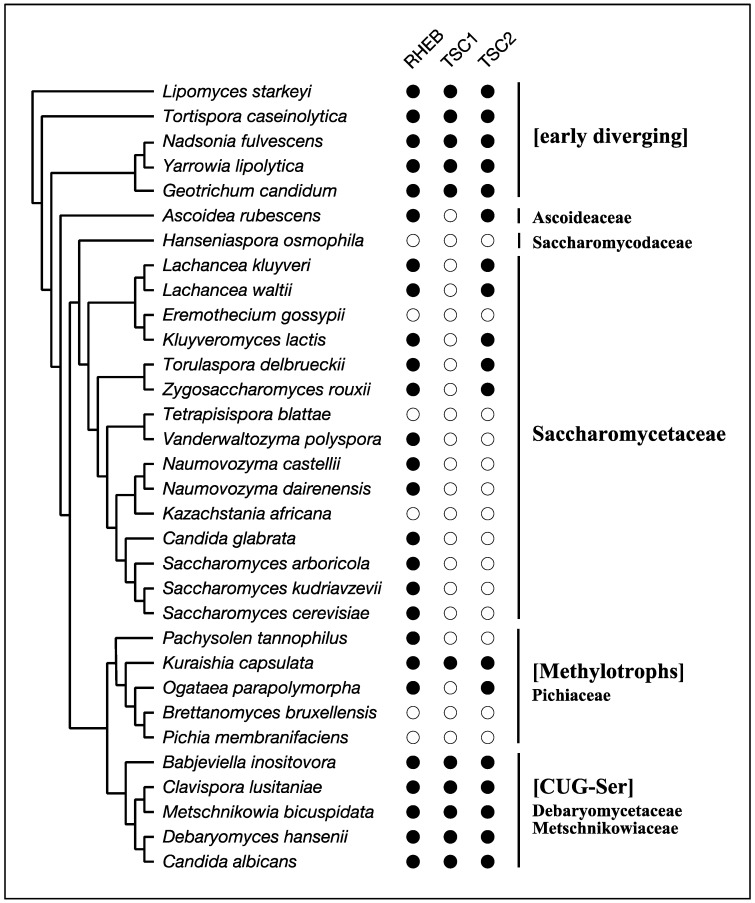
An occurrence chart of RHEB, TSC1 and TSC2 and the consensus cladogram of the subphylum Saccharomycotina. The consensus cladogram was constructed according to the literature [59,61,62]. Occurrence was determined by homology searches with the NCBI BLAST program and by domain searches with the HMMER3 suite (http://hmmer.org) and the Pfam database [63]. Filled circles indicate presence; open circles indicate absence.

**Figure 5:**
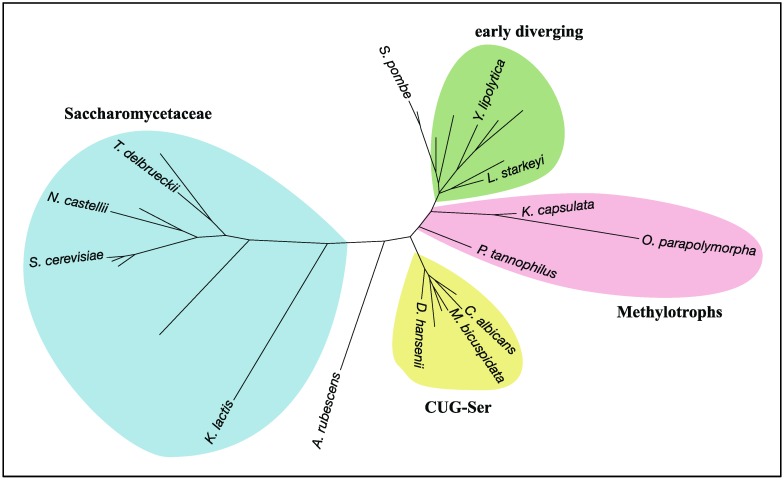
A radial phylogram of RHEB in the subphylum Saccharomycotina. Two species of the subphylum Taphrinomycotina, *Schizosaccharomyces pombe* and *Saitoella complicata*, were also included to clarify the root of Saccharomycotina in the phylogram. The phylogenic tree was constructed by the ETE3 toolkit [64] with the workflow option being “standard_fasttree”, followed by tree drawing by Dendroscope 3 [65]. Labeling nodes and coloring taxa were done manually. *A. rubescens*: *Ascoidea rubescens*; *C. albicans*: *Candida albicans*; *D. hansenii*: *Debaryomyces hansenii*; *K. capsulata*: *Kuraishia capsulate*; *K. lactis*: *Kluyveromyces lactis*; *L. starkeyi*: *Lipomyces starkeyi*; *M. bicuspidata*: *Metschnikowia bicuspidate*; *N. castellii*: *Naumovozyma castellii*; *O. parapolymorpha*: *Ogataea parapolymorpha*; *P. tannophilus*: *Pachysolen tannophilus*; *S. cerevisiae*: *Saccharomyces cerevisiae*; *S. pombe*: *Schizosaccharomyces pombe*; *T. delbrueckii*: *Torulaspora delbrueckii*; *Y. lipolytica*: *Yarrowia lipolytica*.

The best-known species that lacks RHEB-dependent regulation of TORC1 among Opisthokonta is the budding yeast *Saccharomyces cerevisiae*. Its genome carries a gene encoding a RHEB-like small GTPase, but the gene product does not function as an activator of TORC1 [56,57,58]. This yeast species also lacks genes for TSC1 and TSC2 (Table 1; Figure 4). *S. cerevisiae* belongs to the subphylum Saccharomycotina, which is composed mainly of three major clades, CUG-Ser, Methylotrophs, and Saccharomycetaceae as well as several early diverging members (Figure 4) [59]. RHEB, TSC1, and TSC2 are conserved among the early diverging members, such as *Lipomyces starkeyi* and *Yarrowia lipolytica* (Table 1; Figure 4). Members of the CUG-Ser clade, where the CUG codon is translated to Ser instead of Leu due to genetic changes of the tRNA_CAG_, also possess RHEB, TSC1, and TSC2 (Table 1; Figure 4). In *Candida albicans*, a member of the CUG-Ser clade, RHEB is involved in nitrogen starvation-induced filamentation but dispensable for cellular viability, implying a diminished contribution of RHEB to the regulation of TORC1 in this clade [60]. On the other hand, all members in the Saccharomycetaceae clade partially or completely lack RHEB and the TSC subunits. Moreover, RHEB identified in this clade is substantially divergent from those in other members of Saccharomycotina (Figure 5). Such divergence in Saccharomycetaceae implies that RHEB had lost its role in the regulation of TORC1 during early evolution of Saccharomycetaceae and carries out different cellular functions. The Methylotrophs clade also exhibits sporadic loss of RHEB, TSC1, or TSC2 (Figure 4), and how TORC1 is regulated in the absence of RHEB, TSC1, or TSC2 in the Methylotrophs species remains unknown.

Extensive surveys of genome databases demonstrate that RHEB and TSC2 are present in multiple taxa outside the Opisthokonta clade [5] (Table 1). In contrast, TSC1 is not readily identifiable due to its limited sequence conservation among distantly related species [5] (Table 1). In Amoebozoa, while only RHEB and TSC2 can be found in *Dictyostelium* species, *Acanthamoeba castellanii* has all of RHEB, TSC1, and TSC2 (Table 1), suggesting that certain Amoebozoa species possess the intact RHEB-TSC system. None of the Excavata species, such as *Trypanosoma* and *Giardia*, exhibit unequivocal presence of RHEB or the TSC subunits (Table 1). The SAR clade is a huge taxon that includes extremely diverse species [4]. In this clade, the water mold *Aphanomyces invadans* has all of RHEB, TSC1, and TSC2, and multiple other water molds, such as *Pythium irregulare* and *Phytophthora infestans*, possess at least RHEB and TSC2 (Table 1), suggesting that water mold species have the functional RHEB-TSC system. Among Plantae, green algae and land plants have completely lost both RHEB and the TSC subunits, while red algae Rhodophyta appears to retain at least a part of the RHEB-TSC system (Table 1). Appearance of RHEB and TSC2 in such a wide variety of eukaryotes suggests that the RHEB-TSC system arose with TORC1 in LECA but was lost during evolution in multiple taxa, resulting in sporadic occurrence among eukaryotic taxa (Table 1). Obligate, intracellular parasitic species without TOR kinase, such as *Plasmodium falciparum* and *Encephalitozoon intestinalis*, also lack both RHEB and the TSC subunits, suggesting that the primary function of RHEB and the TSC complex is to regulate TORC1 [5].

Assuming that RHEB and the TSC complex played a key role in controlling TORC1 activity in LECA, how have those key regulators been lost in certain species like *S. cerevisiae* where TORC1 activity remains physiologically crucial? In the fission yeast *S. pombe*, RHEB becomes dispensable when TOR kinase carries an activating mutation [53]. It is, therefore, conceivable that mutation(s) activating TOR kinase arose in the common ancestor of the Saccharomycetaceae clade and hence RHEB became less and less important for TORC1 activity in the descendants. However, it is unclear if TOR kinase is intrinsically more active in those RHEB-less *Saccharomyces* species. In mammals and certain other species, it is widely accepted that RHEB and the TSC complex are the key regulatory factors funneling a variety of stimuli to strictly control TORC1 activity. Considering the vital roles of RHEB and the TSC complex in modulating TORC1 activity, it is of great interest how TORC1 remains highly responsive to stimuli in species that lack the RHEB-dependent regulation, such as *S. cerevisiae*.

## 5. The RAG GTPases and Their GAP Complex GATOR1

As mentioned above, mammalian TORC1 is activated by multiple stimuli such as growth factors, cellular energy levels, and nutrients [1,12]. While most input signals modulate the function of the TSC complex and the activity of RHEB to control TORC1, amino acids stimulate TORC1 activity even in the absence of the TSC complex [57,66]. Amino acid stimuli first induce translocation of cytosolic TORC1 to lysosomes, where GTP-bound, active RHEB resides and interacts physically with TORC1 for its activation. The lysosomal translocation of TORC1 is mediated by physical interaction with the RAG small GTPases, which are members of the RAS super-family [67] (Figure 3). Humans have four RAG genes encoding RAG-A, RAG-B, RAG-C, and RAG-D, of which RAG-A and RAG-B form a heterodimer with either RAG-C or RAG-D. The RAG heterodimer is always at the lysosomal surface, but its guanine-nucleotide binding state is responsive to amino acid stimuli [67]. Upon the stimuli, the RAG heterodimer physically binds TORC1 for its recruitment, most efficiently with RAG-A or RAG-B being in the GTP-bound form and RAG-C or RAG-D in the GDP-bound form [67]. The guanine-nucleotide binding state of RAG-A and RAG-B is modulated by a GAP complex called GAP activity towards RAGs 1 (GATOR1), a trimeric protein complex composed of the catalytic DEP domain containing 5 (DEPDC5) subunit and the two regulatory subunits nitrogen permease regulator 2-like protein (NPRL2) and nitrogen permease regulator 3-like protein (NPRL3) [68] (Figure 3). More details about how the RAG GTPase heterodimer and its regulators control TORC1 activity in response to amino acid stimuli are described in other articles in this issue of *Biomolecules* [57,66,69].

The budding yeast *S. cerevisiae* also possesses both the RAG GTPases and the trimeric GATOR1 complex (Table 1). Moreover, it has been reported that, in response to nutritional stimuli, budding yeast RAG and GATOR1 promote TORC1 activity even without RHEB-dependent activation of TORC1 [70,71,72,73,74]. As has been found in mammals, the RAG heterodimer exhibits higher affinity to TORC1 when RAG-A is bound to GTP [71,74]. Both RAG GTPases and TORC1 always reside at the surface of vacuoles (yeast equivalent of mammalian lysosomes) in budding yeast, but nutritional stimuli affect the nucleotide binding state of the RAG heterodimer, altering the pattern of TORC1 distribution on the vacuolar surface [70,74]. It is likely that such a change in TORC1 localization is a part of the mechanism of how the RAG heterodimer activates TORC1 independently of RHEB in budding yeast. Details of the mechanism, however, have to wait for future studies.

Thorough database searches have revealed that the RAG heterodimer and the trimeric GATOR1 complex distribute much more ubiquitously than RHEB and the TSC subunits among the eukaryotic taxa (Table 1). In fact, no taxon that apparently lacks RAG and GATOR1 possesses RHEB and the TSC subunits. Our searches also show that intracellular parasitic species, such as *Plasmodium falciparum* and *Encephalitozoon intestinalis*, have lost RAG and GATOR1 as well as TORC1 (Table 1). Collectively, it is surmised that RAG and GATOR1 arose with TORC1 in LECA and have been functioning for TORC1 regulation since then. Except for species that extremely diverged from other eukaryotes, such as *Giardia intestinalis* [75], the only taxon that evidently lacks RAG and GATOR1 is the green algae and land plant clade. Thus, it is very likely that RAG and GATOR1 was lost in this clade during early evolution. Since the green algae and land plants also lack RHEB and the TSC subunits, they have probably evolved TORC1 regulatory mechanisms that are completely different from those of other species [76]. Like certain animals and fungi, there are taxa that retain RAG and GATOR1 as well as RHEB and the TSC subunits. Such species include *Acanthamoeba castellanii* and *Dictyostelium discoideum* in Amoebozoa, *Plasmodiophora brassicae* in Rhizaria, multiple water mold species such as *Aphanomyces invadans* in Stramenopiles, and red algae *Cyanidioschyzon melorae* and *Galdieria sulphuraria* in Rhodophyta (Table 1). There are, however, also multiple taxa that possess the RAG heterodimer and GATOR1 but lack RHEB or the TSC subunits (Table 1), including *Trypanosoma* species in Excavata, the foram *Reticulomyxa filosa* and the oceanic unicellular algae *Bigelowiella natans* in Rhizaria, and free-living ciliates such as *Paramecium tetraurelia* and *Tetrahumena thermophila* in Alveolata. Frequent appearance of the RAG GTPases without RHEB implies that the RAG GTPases have an evolutionarily conserved function in TORC1 regulation independent of RHEB. Possibly, such a regulatory mechanism is cryptic in mammals where RHEB is absolutely required for TORC1 activation. The budding yeast *S. cerevisiae* and other species that lack the RHEB-dependent TORC1 activation mechanism may provide a useful platform to explore the evolutionarily conserved molecular function of the RAG GTPases.

## Figures and Tables

**Table 1:** Appearance of target of rapamycin (TOR) signaling components among eukaryotic species.

Major Clade	Kingdom	Species	RICTOR	SIN1	TOR	LST8	RAPTOR	RHEB	TSC1	TSC2	RAG-A/B	RAG-C/D	DEPDC5	NPRL2	NPRL3
Opisthokonta	Metazoa	*Homo sapiens*	✔	✔	✔	✔	✔	✔	✔	✔	✔	✔	✔	✔	✔
Opisthokonta	Metazoa	*Ciona intestinalis*	✔	✔	✔	✔	✔	✔	✔	✔	✔	✔	✔	✔	✔
Opisthokonta	Metazoa	*Drosophila melanogaster*	✔	✔	✔	✔	✔	✔	✔	✔	✔	✔	✔	✔	✔
Opisthokonta	Metazoa	*Schistosoma mansoni*	✔	✔	✔	✔	✔				✔	✔	✔	✔	✔
Opisthokonta	Metazoa	*Caenorhabditis elegans*	✔	✔	✔	✔	✔	✔			✔	✔	✔	✔	✔
Opisthokonta	Metazoa	*Nematostella vectensis*	✔	✔	✔	✔	✔	✔	✔	✔	✔	✔	✔	✔	✔
Opisthokonta	Metazoa	*Trichoplax adhaerens*	✔	✔	✔	✔	✔	✔	✔	✔	✔	✔	✔	✔	✔
Opisthokonta		*Amphimedon queenslandica*	✔	✔	✔	✔	✔	✔	✔	✔	✔	✔	✔	✔	✔
Opisthokonta		*Capsaspora owczarzaki*	✔	✔	✔	✔	✔	✔	✔	✔	✔	✔	✔	✔	✔
Opisthokonta	Fungi	*Mitosporidium daphniae*			✔	✔	✔								
Opisthokonta	Fungi	*Encephalitozoon intestinalis*													
Opisthokonta	Fungi	*Rozella allomycis*	✔	✔	✔	✔	✔				✔	✔	✔	✔	✔
Opisthokonta	Fungi	*Allomyces macrogynus*	✔	✔	✔	✔	✔				✔	✔	✔	✔	✔
Opisthokonta	Fungi	*Batrachochytrium dendrobatidis*	✔	✔	✔	✔	✔	✔	✔	✔	✔	✔	✔	✔	✔
Opisthokonta	Fungi	*Gonapodya prolifera*	✔	✔	✔	✔	✔	✔		✔	✔	✔	✔	✔	✔
Opisthokonta	Fungi	*Conidiobolus coronatus*	✔	✔	✔	✔	✔	✔	✔	✔	✔	✔	✔	✔	✔
Opisthokonta	Fungi	*Smittium culicis*	✔	✔	✔	✔	✔	✔		✔	✔	✔	✔	✔	✔
Opisthokonta	Fungi	*Rhizophagus irregularis*	✔	✔	✔	✔	✔	✔	✔	✔	✔	✔	✔	✔	✔
Opisthokonta	Fungi	*Lobosporangium transversale*	✔	✔	✔	✔	✔	✔	✔	✔	✔	✔	✔	✔	✔
Opisthokonta	Fungi	*Mortierella elongata*	✔	✔	✔	✔	✔	✔	✔	✔	✔	✔	✔	✔	✔
Opisthokonta	Fungi	*Mucor circinelloides*	✔	✔	✔	✔	✔	✔	✔	✔	✔	✔	✔	✔	✔
Opisthokonta	Fungi	*Neocallimastix californiae*	✔	✔	✔	✔	✔	✔	✔	✔	✔	✔	✔	✔	✔
Opisthokonta	Fungi	*Puccinia sorghi*	✔	✔	✔	✔	✔	✔	✔	✔	✔	✔	✔	✔	✔
Opisthokonta	Fungi	*Schizophyllum commune*	✔	✔	✔	✔	✔	✔	✔	✔	✔	✔	✔	✔	✔
Opisthokonta	Fungi	*Cutaneotrichosporon oleaginosus*	✔	✔	✔	✔	✔	✔	✔	✔	✔	✔	✔	✔	✔
Opisthokonta	Fungi	*Tilletiaria anomala*	✔	✔	✔	✔	✔	✔	✔	✔	✔	✔	✔	✔	✔
Opisthokonta	Fungi	*Ustilago maydis*	✔	✔	✔	✔	✔	✔	✔	✔	✔	✔	✔	✔	✔
Opisthokonta	Fungi	*Dothistroma septosporum*	✔	✔	✔	✔	✔	✔	✔	✔	✔	✔	✔	✔	✔
Opisthokonta	Fungi	*Cladophialophora bantiana*	✔	✔	✔	✔	✔	✔		✔	✔	✔	✔	✔	✔
Opisthokonta	Fungi	*Aspergillus fumigatus*	✔	✔	✔	✔	✔	✔		✔	✔	✔	✔	✔	✔
Opisthokonta	Fungi	*Trichopyton equinum*	✔	✔	✔	✔	✔	✔		✔	✔	✔	✔	✔	✔
Opisthokonta	Fungi	*Botrytis cinerea*	✔	✔	✔	✔	✔	✔	✔	✔	✔	✔	✔	✔	✔
Opisthokonta	Fungi	*Colletotrichum graminicola*	✔	✔	✔	✔	✔	✔	✔	✔	✔	✔	✔	✔	✔
Opisthokonta	Fungi	*Fusarium fujikuroi*	✔	✔	✔	✔	✔	✔	✔	✔	✔	✔	✔	✔	✔
Opisthokonta	Fungi	*Neurospora crassa*	✔	✔	✔	✔	✔	✔	✔	✔	✔	✔	✔	✔	✔
Opisthokonta	Fungi	*Schizosaccharomyces pombe*	✔	✔	✔	✔	✔	✔	✔	✔	✔	✔	✔	✔	✔
Opisthokonta	Fungi	*Saitoella complicata*	✔	✔	✔	✔	✔	✔	✔	✔	✔	✔	✔	✔	✔
Opisthokonta	Fungi	*Yarrowia lipolytica*	✔	✔	✔	✔	✔	✔	✔	✔	✔	✔	✔	✔	✔
Opisthokonta	Fungi	*Candida albicans*	✔	✔	✔	✔	✔	✔	✔	✔	✔	✔	✔	✔	✔
Opisthokonta	Fungi	*Ogataea parapolymorpha*	✔	✔	✔	✔	✔	✔		✔	✔	✔	✔	✔	✔
Opisthokonta	Fungi	*Eremothecium gossypii*	✔	✔	✔	✔	✔				✔	✔	✔	✔	✔
Opisthokonta	Fungi	*Kluyveromyces lactis*	✔	✔	✔	✔	✔	✔		✔	✔	✔	✔	✔	✔
Opisthokonta	Fungi	*Candida glabrata*	✔	✔	✔	✔	✔				✔	✔	✔	✔	✔
Opisthokonta	Fungi	*Saccharomyces cerevisiae*	✔	✔	✔	✔	✔	✔			✔	✔	✔	✔	✔
Amoebozoa		*Acanthamoeba castellanii*	✔	✔	✔	✔	✔	✔	✔	✔	✔	✔		✔	
Amoebozoa		*Dictyostelium discoideum*	✔	✔	✔	✔	✔	✔		✔	✔	✔	✔	✔	✔
Amoebozoa		*Entamoeba histolytica*	✔		✔		✔				✔	✔	✔	✔	✔
Excavata		*Naegleria gruberi*	✔	✔	✔	✔	✔				✔	✔	✔	✔	✔
Excavata		*Bodo saltans*	✔	✔	✔	✔ ^1^	✔				✔	✔	✔	✔	✔
Excavata		*Angomonas deanei*	✔	✔	✔	✔ ^1^	✔				✔	✔	✔		
Excavata		*Trypanosoma brucei*	✔		✔	✔ ^1^	✔				✔	✔	✔	✔	
Excavata		*Leishmania major*	✔	✔	✔	✔ ^1^	✔				✔	✔	✔		
Excavata		*Giardia intestinalis*	✔		✔	✔	✔								
Excavata		*Spironucleus salmonicida*	✔		✔	✔	✔								
SAR	Rhizaria	*Reticulomyxa filosa*	✔	✔	✔	✔	✔				✔	✔	✔	✔	✔
SAR	Rhizaria	*Plasmodiophora brassicae*	✔	✔	✔	✔	✔	✔		✔	✔	✔	✔	✔	✔
SAR	Rhizaria	*Bigelowiella natans*	✔	✔	✔	✔	✔				✔	✔	✔	✔	✔
SAR	Alveolata	*Stylonychia lemnae*	✔	✔	✔	✔					✔	✔	✔	✔	✔
SAR	Alveolata	*Oxytricha trifallax*	✔	✔	✔	✔					✔	✔	✔	✔	✔
SAR	Alveolata	*Paramecium tetraurelia*	✔	✔	✔	✔					✔	✔	✔	✔	
SAR	Alveolata	*Tetrahymena thermophila*	✔	✔	✔	✔					✔	✔	✔	✔	✔
SAR	Alveolata	*Vitrella brassicaformis*	✔	✔	✔	✔	✔				✔	✔	✔	✔	✔
SAR	Alveolata	*Plasmodium falciparum*													
SAR	Alveolata	*Hammondia hammondi*			✔						✔	✔			
SAR	Alveolata	*Toxoplasma gondii*			✔						✔	✔			
SAR	Alveolata	*Cyclospora cayetanensis*													
SAR	Alveolata	*Eimeria maxima*													
SAR	Alveolata	*Cryptosporidium pavrum*													
SAR	Alveolata	*Theileria annulata*													
SAR	Stramenopiles	*Aureococcus anophagefferens*			✔	✔	✔	✔			✔	✔			
SAR	Stramenopiles	*Saprolegnia diclina*	✔	✔	✔	✔	✔	✔		✔	✔	✔	✔	✔	✔
SAR	Stramenopiles	*Aphanomyces invadans*	✔	✔	✔	✔	✔	✔	✔	✔	✔	✔	✔	✔	✔
SAR	Stramenopiles	*Pythium irregulare*	✔	✔	✔	✔	✔	✔		✔	✔	✔	✔	✔	✔
SAR	Stramenopiles	*Phytophthora infestans*	✔	✔	✔	✔	✔	✔		✔	✔	✔	✔	✔	✔
SAR	Stramenopiles	*Plasmopara halstedii*	✔	✔	✔	✔	✔	✔	✔		✔	✔	✔	✔	✔
SAR	Stramenopiles	*Hyaloperonospora arabidopsis*	✔	✔	✔	✔	✔	✔		✔	✔	✔	✔	✔	✔
SAR	Stramenopiles	*Albugo candida*	✔	✔	✔	✔	✔	✔		✔	✔	✔	✔	✔	✔
SAR	Stramenopiles	*Nannochloropsis gaditana*		✔	✔	✔	✔	✔			✔	✔	✔	✔	✔
SAR	Stramenopiles	*Blastocystis hominis*	✔		✔	✔	✔								✔
SAR	Stramenopiles	*Phaeodactylum tricornutum*			✔	✔	✔	✔	✔	✔	✔	✔	✔	✔	
Plantae	Rhodophyta	*Cyanidioschyzon melorae*			✔	✔	✔	✔		✔	✔	✔	✔		
Plantae	Rhodophyta	*Galdieria sulphuraria*			✔	✔	✔	✔			✔	✔	✔	✔	
Plantae	Rhodophyta	*Chondrus crispus*			✔		✔	✔							
Plantae	Chlorophyta	*Ostreococcus lucimarinus*			✔	✔	✔								
Plantae	Streptophyta	*Selaginella moellendorffii*			✔	✔	✔								
Plantae	Streptophyta	*Amborella trichopoda*			✔	✔	✔								
Plantae	Streptophyta	*Oryza sativa*			✔	✔	✔								
Plantae	Streptophyta	*Arabidopsis thaliana*			✔	✔	✔								

^1^ A *Trypanosoma brucei* protein (NCBI: XP_828034) was experimentally determined as a lethal with sec thirteen 8 (LST8) ortholog, although this protein shows only limited similarity to human and yeast LST8 [6]. The LST8 orthologs in *Leishmania major*, *Bodo saltans*, and *Angomonas deanei* were identified by the NCBI BLAST program with *Trypanosoma brucei* LST8 as a query. DEPDC5: DEP domain containing 5; NPRL2: nitrogen permease regulator 2-like protein; NPRL3: nitrogen permease regulator 3-like protein; RAPTOR: regulatory associated protein of mTOR; RHEB: Ras homolog enriched in brain; RICTOR: rapamycin-insensitive companion of mTOR; SIN1: stress-activated protein kinase interacting protein 1; TOR: target of rapamycin; TSC1, 2: tuberous sclerosis complex 1, 2.
